# Tool report: EvoMaster—black and white box search-based fuzzing for REST, GraphQL and RPC APIs

**DOI:** 10.1007/s10515-024-00478-1

**Published:** 2024-11-29

**Authors:** Andrea Arcuri, Man Zhang, Susruthan Seran, Juan Pablo Galeotti, Amid Golmohammadi, Onur Duman, Agustina Aldasoro, Hernan Ghianni

**Affiliations:** 1 School of Economics, Innovation, and Technology, Kristiania University College, Kirkegata 24-26, 0153 Oslo, Norway; 2 Department of Computer Science, Oslo Metropolitan University, Pilestredet 35, 0166 Oslo, Norway; 3 Beihang University, Beijing, China; 4 University of Buenos Aires, Buenos Aires, Argentina

**Keywords:** Fuzzing, SBST, Web API, Tool

## Abstract

In this paper, we present the latest version 3.0.0 of EvoMaster, an open-source search-based fuzzer aimed at Web APIs. We discuss and present all its recent improvements, including advanced white-box heuristics, advanced search algorithms, support for databases and external services, as well as dealing with GraphQL and RPC APIs besides the original use case for REST APIs. The tool’s installers have been downloaded more than 3000 times. EvoMaster is in daily use for fuzzing millions of lines of code in hundreds of APIs in large Fortune 500 companies, such as for example the e-commerce Meituan.

## Introduction

Web services, and in particular RESTful APIs, are widespread in industry, providing rich APIs available on the internet. Thousands of Web APIs exist.^1^^,^^2^ Besides providing functionality over the internet, this kind of APIs are often used to build *microservice architectures* (Newman 2021; Rajesh 2016). Testing Web APIs is challenging and expensive in industry (Arcuri 2018b). As such, viable automated techniques to reduce cost and improve test effectiveness are needed.

EvoMaster is a mature search-based tool aimed at test case generation for system testing. It has been developed based on the lessons learned from our previous tool EvoSuite (Fraser and Arcuri 2011), aimed at unit test generation for Java classes. EvoMaster is open-source (Arcuri et al. 2021) hosted on GitHub,^3^ and it has been under development since 2016. EvoMaster was originally designed to perform white-box fuzzing for REST APIs (Arcuri 2017b, 2019), and used for designing and evaluating novel search algorithms such as MIO (Arcuri 2017a, 2018c). In 2018, a tool paper (Arcuri 2018a) presented the technical work and usage of EvoMaster. However, since then, a lot of work has been done to extend EvoMaster in different directions and improve its usability for practitioners in industry.

In this paper, we present the newest version of EvoMaster, namely version 3.0.0, released on GitHub and Zenodo (Arcuri et al. 2024). We provide a brief summary of all the major features that have been added in the last few years. Of particular interest is its usage by other researchers and among practitioners in industry.

## The tool

**Fig. 1:**
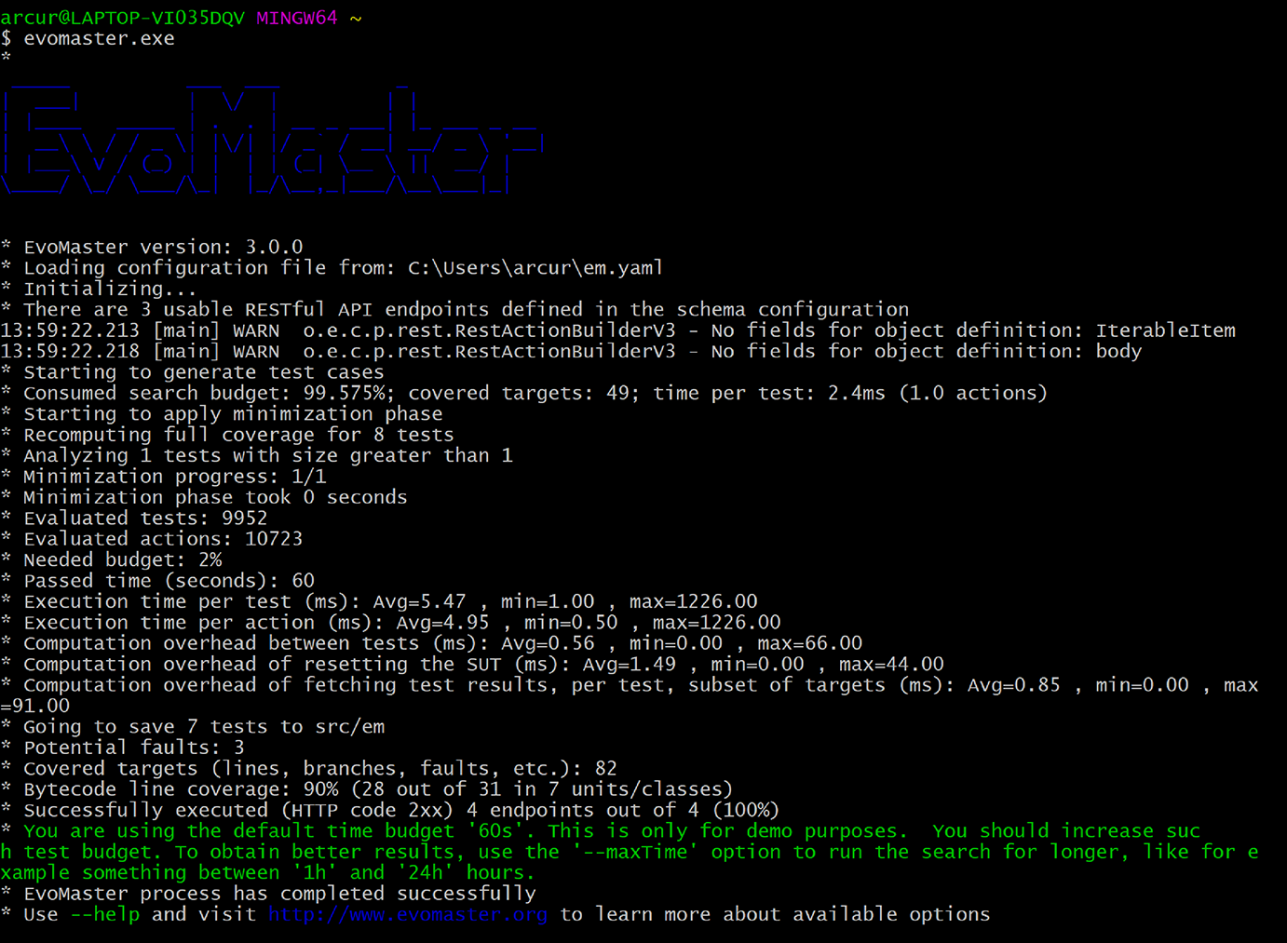
Screenshot of command-line execution of EvoMaster on a sample API

EvoMaster is currently a command-line tool, built with Kotlin and Java. Figure 1 shows an example of its usage. Each new release has installer files for all the major operating systems (i.e., Windows, OSX and Linux). EvoMaster can be run in two different modes (Arcuri 2020; Martin-Lopez et al. 2021a): *black-box* and *white-box*.^4^ The black-box mode is easier to use, as it just requires the API being up and running and having access to its defining schema. As no code analysis is done, the API could be implemented in any programming language. Also, the API could be remote on the internet. On the other hand, white-box testing requires access to the running process of the API, to allow runtime instrumentation. Furthermore, it requires the user to manually write a “driver”/configuration file to specify how to start, stop and reset the API. White-box testing is harder to set up and it is of more narrow scope (as the instrumentation is programming-language dependent), but can provide much better results (Arcuri 2020; Zhang and Arcuri 2023), e.g., in terms of code coverage and fault detection. To the best of our knowledge, EvoMaster is currently the only open-source fuzzer for Web APIs that supports white-box testing (Golmohammadi et al. 2023b).

The output of the tool is executable test cases (e.g., in JUnit format). Different kinds of automated oracles are used to detect faults (Marculescu et al. 2022) (e.g., server crashes leading to responses with HTTP status code 500).

To fuzz an API, EvoMaster needs to get as input a specification for it. This is needed to know what can be called on the API, and what types of inputs it accepts. Sending random bytes over a TCP connection will likely result in invalid messages that the API would directly discard. For REST APIs, schemas are typically defined with the OpenAPI format. For GraphQL, the schema can be queried directly from the API via an introspective query. Different RPC frameworks (e.g., gRPC and Thrift) use different DSLs to specify the schema (e.g., protobuf for gRPC). But, ultimately, most RPC frameworks enable the generation of client libraries from the schema to be able to call the API programmatically.

**Fig. 2:**
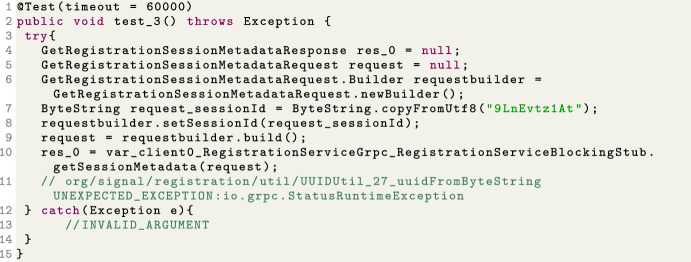
Example of generated JUnit test for the gRPC *signal-registration* API. For reasons of space, the code has been slightly formatted

Let us consider the example of fuzzing the *signal-registration* gRPC API (part of the backend of the popular communication app called *Signal*), which is now included in the EMB corpus (Arcuri et al. 2023b). EvoMaster can generate test cases like the one shown in Fig. 2. Here, the remote procedure call fails with an exception, as one of the inputs is a sequence of bytes (represented with the ByteString type), although internally the API expects it to represent a valid UUID. This is an unexpected exception from the point of view of the schema of this API, revealing so a fault.

## Enhancements in EvoMaster 3.0.0

EvoMaster is actively maintained, with researchers from Norway, China and Argentina regularly working on it. Here, we provide a short summary of all the major features added to EvoMaster in the recent years (not in chronological order) since the previous tool report in 2018 (Arcuri 2018a). The interested reader is referred to the cited articles for more details on these features. These features include enhancements in the tool’s search engine (i.e., search-algorithms and white-box heuristics), its application on different programming languages, specific heuristics for REST APIs, handling of the APIs’ environment such as databases and external services, as well as its support for other kinds of web services such as GraphQL and RPC.

*Search-algorithms* When addressing a new problem with search algorithms, like for example system test generation for Web APIs, there is the opportunity to design novel algorithms that exploit as much domain knowledge as possible. This can lead to better results for that specific problem domain. That was one of the original motivations for designing MIO (Arcuri 2017a, 2018c).

One major improvement over the original MIO was the introduction of *adaptive hypermutation* (Zhang and Arcuri 2021a). Due to the massive search space (e.g., when thinking about sequences of HTTP calls, with complex JSON body payloads), where possibly many parts of the genotype have no impact on the phenotype (e.g., input data that is simply stored into a database, with no influence on the execution control flow of the tested API), a higher mutation rate might be beneficial. This is handled adaptively, based on the search phase (e.g., mutation rate decreasing throughout the search), and based on the feedback from the fitness function for each mutated gene.

*White-box heuristics* The performance of a search algorithm is strongly dependent on the used fitness function. In Search-Based Software Testing (SBST) research, work has been done to improve the fitness function to achieve higher code coverage, e.g., using standard techniques like the *branch distance*, for numerical and string data. EvoMaster uses these common techniques from the SBST literature (e.g., like EvoSuite). Furthermore, we designed novel techniques based on *testability transformations* (Arcuri and Galeotti 2021, 2020b), in particular for dealing with JDK API calls.

One of the challenges when fuzzing REST APIs is that their defining schema (e.g., in OpenAPI format) might be underspecified. For example, if the existence of a URL query parameter is not specified in the schema, a black-box fuzzer would have no information on how to use it. However, when doing white-box testing, dynamic analyses of the API can detect these missing cases, which can then be used to improve the fuzzing (Arcuriet al. 2023a).

*Language support* Black-box testing can be applied on any web application accessed through API calls, regardless of its programming language (e.g., Go and Python). However, white-box testing is dependent on the programming language, as code needs to be analyzed. Since its inception, EvoMaster has been focusing on the JVM, in particular on languages such as Java and Kotlin.

The JVM is widely used in industry, but there are other languages/runtimes that are widely popular as well for developing Web APIs, like for example NodeJS and .NET. To make EvoMaster more popular among practitioners, we have carried out work to support white-box fuzzing for NodeJS (Zhang et al. 2022b, 2023b) (JavaScript and TypeScript) and .NET (Golmohammadi et al. 2023a) (C#) APIs.

Unfortunately, supporting different programming languages for white-box testing is a gargantuan task, which we found out that most researchers consider only as technical work. Therefore, such line of research has been discontinued. For the time being, the support for NodeJS and .NET in EvoMaster can be considered just as an academic proof-of-concept.

*REST resources* To test a REST API, there might be the need to create some data first (e.g., with an HTTP POST request) before being able to test a fetch method (e.g., an HTTP GET request). Likewise, you need to have some data first before being able to test other kinds of operations such as delete and update. How read and write operations are related to the same resources is not necessarily obvious, as each operation could be handled by different HTTP endpoints.

If an API follows proper REST guidelines, it is possible to infer relations (e.g., dependencies) among endpoints based on the schema. This information can be exploited by the search algorithms to improve performance when evolving test cases (Zhang et al. 2019, 2021). Relations can also be inferred based on what each endpoint accesses in the databases (Zhang and Arcuri 2021b).

*Databases* Web APIs typically interact with databases, like for example Postgres and MySQL. The execution flow of the API can depend on what returned from the SQL SELECT commands when retrieving data. But these commands could have complex constraints, e.g., in the WHERE clauses. Before thoroughly testing a GET endpoint, there might be the need to first create the right data with POST or PUT requests. The fitness function of search algorithms in EvoMaster has been extended to take into account the constraints in these SQL commands, to help creating the right test data (Arcuri and Galeotti 2020a, 2019).

A further issue is that the data in the database could be “read-only” for the API, e.g., the data could be created by other services or scheduled tasks/scripts. There might be no HTTP method to create the needed data to test retrieve operations. To solve this issue, EvoMaster is currently able to inject data directly into the SQL databases (Arcuri and Galeotti 2020a, 2019). Database initialization data will be evolved like any other element in the test cases, like HTTP query parameters and JSON body payloads.

*External services* It is common, especially in microservice architectures, that an API communicates with other APIs to be able to fulfill its functionalities. For testing, this is problematic, as communications with external services are a source of non-determinism, which can lead to flaky tests. For example, those external services could return different data at each call, or become temporarily unavailable all of a sudden. Furthermore, it would be hard to test specific scenarios (especially error-related ones) if the tester does not have full control of these external services. This is a common problem in industry, where a typical solution is to use *mocking* (e.g., with popular libraries such as WireMock for JVM, to stub HTTP servers used to simulate those external services).

In EvoMaster, we have initial support to automatically instantiate WireMock servers to mock communications with external services (Seran et al. 2023). How to setup these instances (e.g., how to create JSON payloads in their responses) becomes part of the search process. The generated tests are then able to start those WireMock instances, configured with the evolved data in their HTTP responses.

*GraphQL APIs* REST is only one kind of Web APIs, albeit arguably the most popular. An alternative approach for Web APIs is GraphQL (Quiña-Mera et al. 2023), originally introduced by Facebook/Meta. Typical GraphQL APIs provide a single HTTP endpoint where data can be fetched and manipulated via a graph-based query language.

EvoMaster has been extended to be able to fuzz GraphQL APIs (Belhadi et al. 2023). Several components of EvoMaster discussed in Sect. 3 could be reused, e.g., search algorithms, white-box heuristics, and database support.

However, research was needed to define how fuzzing GraphQL could be effectively cast to a search problem, and how to define proper automated oracles for this testing domain (Belhadi et al. 2023).

*RPC APIs* Besides REST and GraphQL APIs, another common type of APIs is Remote Procedure Call (RPC) ones. Popular examples in industry are gRPC (from Google/Alphabet) and Thrift (originally from Facebook/Meta). Albeit less popular for APIs available on the internet, RPC are very common in enterprise backends when using microservice architectures.

EvoMaster is currently supporting all different kinds of RPC frameworks (Zhang et al. 2023a) (e.g., gRPC and Thrift), as long as a client library is provided (which is a typical case for RPC frameworks). Supporting RPC was mainly driven by an industry collaboration with Meituan (Zhanget al. 2022a), a Fortune 500 large e-commerce Chinese enterprise with more than 600 million customers. Similar to GraphQL support, most of the internal features of EvoMaster could be re-used to address this new problem domain. Albeit their popularity in industry, to the best of our knowledge currently EvoMaster is the only tool that supports the fuzzing of this type of APIs, besides (Veldkamp et al. 2023).

## Usage by other researchers

In the literature, besides by its authors, EvoMaster has been used in several studies. A typical example is tool comparisons (e.g., Kim et al. 2022, 2023a, b; Liu et al. 2022; Giamattei et al. 2023; Karlsson et al. 2023). Another example involves the studying of carving UI tests to generate API tests (Yandrapally et al. 2023).

As EvoMaster is open-source, different authors have extended it to address different research questions. Examples include handling domain-specific coverage (Laaber et al. 2023), applications of hierarchical clustering (Stallenberg et al. 2021) and studying of Artificial Bee Colony optimization algorithms (Sahin and Akay 2021).

## Usage in industry

At the time of writing, EvoMaster has more than 500 stars on GitHub. According to the download statistics of GitHub, its installer files have been downloaded more than 3000 times. However, this does not include possible users that fork its repository or simply download it with Git and build EvoMaster locally.

As part of industry-driven research, we collaborate with different enterprises. An example is Meituan (Zhanget al. 2022a), previously discussed in Sect. 3 regarding RPC support. Currently, EvoMaster is integrated into their development and testing processes. Hundreds of engineers at Meituan reap the benefits of EvoMaster daily, where it is used to white-box fuzz hundreds of different RPC APIs in their Continuous Integration systems. Several faults have been automatically found using EvoMaster. Another more recent example is Volkswagen (another Fortune 500 enterprise, which is one of the largest car manufacturers in the world), where EvoMaster has been recently started to be used for black-box fuzzing some of their REST APIs.

Note: these two different enterprises are just examples of direct collaborations, where we are in direct contact each month with the testers and developers there to get their feedback on the use of EvoMaster. We currently do not have data on how many other enterprises in the world are actively using EvoMaster, besides what we can infer from download statistics and from the profile (e.g., GitHub and LinkedIn) of the engineers that report bugs or ask for feature requests. Based on this profile data that we checked, we can see a moderate interest among practitioners in industry.

## Related work

In the last few years, several techniques have been developed to automatically test REST APIs (Golmohammadi et al. 2023b). Several tools in the literature exist, for example (in alphabetic order): bBOXRT (Laranjeiro et al. 2021), Dredd,^5^ Fuzz-lightyear,^6^ Morest (Liu et al. 2022), ResTest (Martin-Lopez et al. 2021b), RestCT (Wu et al. 2022), Restler (Atlidakis et al. 2019), RestTestGen (Viglianisi et al. 2020) Schemathesis (Hatfield-Dodds and Dygalo 2022), and Tcases.^7^ However, to the best of our knowledge, only EvoMaster supports white-box testing (Golmohammadi et al. 2023b). All these other tools support only black-box testing, i.e., generating test cases from the OpenAPI schemas without analyzing the internal code of the tested APIs. Tool comparisons (Kim et al. 2022; Zhang and Arcuri 2023) show that EvoMaster achieves among the best performances (in terms of code coverage and fault detection).

In contrast to REST APIs (Golmohammadi et al. 2023b), the testing of GraphQL and RPC APIs has received only little attention from the research literature (e.g., Karlsson et al. 2020; Zetterlund et al. 2022; Veldkamp et al. 2023), despite their widespread usage in industry.

## Conclusion

In this paper, we presented the latest version 3.0.0 of EvoMaster. EvoMaster is a search-based fuzzer aimed at Web APIs, including REST, GraphQL and RPC. It is a mature open-source tool, under development since 2016. In this paper, we discussed its main features added in the recent years, together with a discussion of its usage among other researchers and practitioners in industry. To the best of our knowledge, it is the only tool in the literature that supports white-box testing in this domain.

There are still many open problems that need to be addressed to achieve better results (Zhang and Arcuri 2023), including defining better white-box heuristics and supporting other features of web services, like dealing with NoSQL databases (e.g., MongoDB). Future work will aim at addressing these issues. Furthermore, as the tool is open-source with copious documentation, it can enable other researchers to use it as starting point for investigating other research directions related to software testing, or related to where test cases are needed to be generated automatically. To learn more about EvoMaster, visit www.evomaster.org.

## Data Availability

EvoMaster is open-source on GitHub,^3^ with each release automatically published on Zenodo (e.g., Arcuri et al. 2024) for long-term storage.

## Footnotes

1 https://apis.guru/.

2 https://rapidapi.com/.

3 https://github.com/WebFuzzing/EvoMaster.

4 https://glossary.istqb.org.

5 https://github.com/apiaryio/dredd.

6 https://github.com/Yelp/fuzz-lightyear.

7 https://github.com/Cornutum/tcases/tree/master/tcases-openapi.
